# Unbiased estimates of cerebrospinal fluid β-amyloid 1–42 cutoffs in a large memory clinic population

**DOI:** 10.1186/s13195-016-0233-7

**Published:** 2017-02-14

**Authors:** Daniela Bertens, Betty M. Tijms, Philip Scheltens, Charlotte E. Teunissen, Pieter Jelle Visser

**Affiliations:** 1 Department of Neurology, Neuroscience Campus Amsterdam, VU University Medical Center, Amsterdam, The Netherlands; 2 Alzheimer Center, Neuroscience Campus Amsterdam, VU University Medical Center, De Boelelaan 1118, 1081, HZ Amsterdam, The Netherlands; 3 Department of Clinical Chemistry, Neurochemistry Lab and Biobank, Neuroscience Campus Amsterdam, VU University Medical Center, Amsterdam, The Netherlands; 4 Alzheimer Center, School for Mental Health and Neuroscience (MHeNS), University Medical Centre Maastricht, Maastricht, The Netherlands

**Keywords:** Alzheimer’s disease, MCI, Cerebrospinal fluid, Diagnosis

## Abstract

**Background:**

We sought to define a cutoff for β-amyloid 1–42 in cerebrospinal fluid (CSF), a key marker for Alzheimer’s disease (AD), with data-driven Gaussian mixture modeling in a memory clinic population.

**Methods:**

We performed a combined cross-sectional and prospective cohort study. We selected 2462 subjects with subjective cognitive decline, mild cognitive impairment, AD-type dementia, and dementia other than AD from the Amsterdam Dementia Cohort. We defined CSF β-amyloid 1–42 cutoffs by data-driven Gaussian mixture modeling in the total population and in subgroups based on clinical diagnosis, age, and apolipoprotein E (APOE) genotype. We investigated whether abnormal β-amyloid 1–42 as defined by the data-driven cutoff could better predict progression to AD-type dementia than abnormal β-amyloid 1–42 defined by a clinical diagnosis-based cutoff using Cox proportional hazards regression.

**Results:**

In the total group of patients, we found a cutoff for abnormal CSF β-amyloid 1–42 of 680 pg/ml (95% CI 660–705 pg/ml). Similar cutoffs were found within diagnostic and APOE genotype subgroups. The cutoff was higher in elderly subjects than in younger subjects. The data-driven cutoff was higher than our clinical diagnosis-based cutoff and had a better predictive accuracy for progression to AD-type dementia in nondemented subjects (HR 7.6 versus 5.2, *p* < 0.01).

**Conclusions:**

Mixture modeling is a robust method to determine cutoffs for CSF β-amyloid 1–42. It might better capture biological changes that are related to AD than cutoffs based on clinical diagnosis.

**Electronic supplementary material:**

The online version of this article (doi:10.1186/s13195-016-0233-7) contains supplementary material, which is available to authorized users.

## Background

Decreased β-amyloid 1–42 (Aβ_42_) in cerebrospinal fluid (CSF) is indicative of Alzheimer’s disease (AD) pathology and part of research criteria for AD [1–4]. However, there is no universal cutoff value to define abnormal CSF Aβ_42_. This is in part due to the variability of Aβ_42_ measurements across laboratories [5, 6]. In addition, clinical centers have used different methods to define a cutoff [7]. Often, a cutoff value is determined by comparing CSF Aβ_42_ levels of cognitively normal subjects with those of patients with a clinical diagnosis of AD-type dementia. However, about 10% of the subjects with clinical AD-type dementia do not have amyloid pathology [8], and 25% of subjects with normal cognition can have amyloid pathology [9], which biases the cutoff value. Data-driven Gaussian mixture modeling provides an alternative approach that does not rely on clinical diagnosis [10]. With this approach, CSF Aβ_42_ levels showed a bimodal distribution representing a normal and an abnormal population. However, it is unclear whether this method is influenced by clinical diagnosis and risk factors for AD.

We aimed to define a cutoff for CSF Aβ_42_ with mixture modeling and to investigate whether this cutoff was dependent on clinical diagnosis, age, and apolipoprotein E (APOE) genotype. We compared the diagnostic accuracy and the predictive accuracy for AD-type dementia progression of the new cutoff value with our previous clinically based cutoff [11]. We also performed a simulation analysis to determine the minimum sample size needed to reliably estimate the cutoff with mixture modeling.

## Methods

### Participants

We selected 2462 subjects from the Amsterdam Dementia Cohort (ADC) [12] with subjective cognitive decline (SCD; *n* = 448), mild cognitive impairment (MCI; *n* = 490), AD dementia (*n* = 1031), and dementia other than AD (*n* = 493) who had CSF measurements available at the time of their first visit at the memory clinic between August 1997 and July 2015. All patients underwent standardized dementia screening at baseline, including physical and neurological examinations, electroencephalograms, magnetic resonance imaging, and laboratory tests. Cognitive screening included the Mini Mental State Examination and, in over 90% of the subjects, a comprehensive neuropsychological test battery. Diagnoses were made by consensus among a multidisciplinary team that did not have knowledge of the CSF results, and they were based on the following clinical criteria. Subjects were diagnosed with SCD when cognitive complaints were present but criteria for MCI, dementia, or any other neurological or psychiatric disorders were not met and all other examinations were normal [13]. Subjects were diagnosed with MCI according to the established MCI criteria [14]. Subjects with AD-type dementia were diagnosed according to the criteria of the National Institute of Neurological and Communicative Disorders and Stroke-Alzheimer’s Disease and Related Disorders Association [4, 15]. Subjects with non-AD-type dementia included subjects with behavioral variants of frontotemporal dementia (*n* = 204) [16, 17], dementia with Lewy bodies (*n* = 113) [18], vascular dementia (*n* = 66) [19], corticobasal degeneration or syndrome (*n* = 31 or *n* = 10, respectively) [20], progressive supranuclear palsy (*n* = 44) [21], alcohol-related dementia (*n* = 3), Huntington’s disease (*n* = 3), Parkinson’s disease (*n* = 1), normal pressure hydrocephalus (*n* = 3), CADASIL (cerebral autosomal dominant arteriopathy with subcortical infarcts and leukoencephalopathy; *n* = 1), Creutzfeldt-Jakob disease (*n* = 3), tauopathy (*n* = 2), and dementia without a known cause (*n* = 9). All subjects gave written informed consent for the use of their clinical and biomarker data for research purposes, and the ethical review board of the VU University Medical Center approved the study.

### Follow-up assessment

Subjects were followed according to clinical needs. The standard follow-up procedure included a 6-month follow-up examination for subjects with dementia and a 12-month follow-up visit for subjects without dementia [12]. Neuropsychological tests were repeated every 12 months. Diagnoses at follow-up were made on the basis of clinical criteria listed above by consensus among a multidisciplinary team.

### CSF Aβ_1–42_ analyses

CSF was collected from 67% of the subjects in the ADC [12]. Reasons for not collecting CSF were refusal, contraindications, technical failure, or CSF having been collected elsewhere. CSF was obtained by lumbar puncture using a 25-gauge needle with a syringe into 10-ml polypropylene tubes (Sarstedt, Nümbrecht, Germany). Within 2 h, CSF samples were centrifuged at 1800 × *g* for 10 minutes at 4 °C. The CSF supernatant was transferred to new polypropylene tubes and stored at −20 °C until further analysis (within 2 months). Baseline Aβ_1–42_ was measured using a commercially available enzyme-linked immunosorbent assay (Innotest β-amyloid(1-_42_; Innogenetics, Ghent, Belgium) on a routine basis as described before [11]. The intra-assay coefficient of variation (mean ± SD) for Aβ_1–42_ was 2.0 ± 0.5%, calculated by averaging the coefficient of variation of duplicates from five runs randomly selected over 2 years. The inter-assay coefficient of variation (mean ± SD) was 10.9 ± 1.8%, as analyzed in a high and low pool from 13 consecutive pool preparations used in total in 189 runs. The team performing the CSF analysis was unaware of the clinical diagnoses.

### Statistical analyses

Baseline characteristics were compared between diagnostic groups with analysis of variance and Kruskal-Wallis or chi-square tests, where appropriate, using IBM SPSS Statistics version 20.0 software (IBM, Armonk, NY, USA). A difference with a *p* value less than 0.05 was considered significant. Gaussian mixture modeling was used to define a cutoff for abnormal CSF Aβ_42_ using the R statistical software program version 3.2.1 mixtools package. First, the number of distributions that best described the data was determined with the R boot.comp function. Next, we defined a data-driven cutoff as the point where the lines of two fitted normal distributions crossed each other. The main analyses included all subjects. We repeated mixture modeling in subgroups based on diagnosis, age (dichotomized based on the median age of 66.5 years), and APOE ε4 allele carriership. Bootstrap sampling was used to determine 95% CIs of the cutoff. Cutoffs were considered to be statistically different between subgroups when their 95% CIs did not overlap.

New cutoff values were compared with our previously clinically defined cutoff of 550 pg/ml, which was based on subjects seen between 2001 and 2007 (*n* = 1070) [11]. For this comparison, we repeated mixture modeling in a subset of data including subjects from this time period. Using this subset, we further tested differences between the clinical and new cutoff values in discrimination between nondemented subjects with or without AD-type dementia at follow-up. In addition, using Cox proportional hazard models, we compared the association of the old cutoff and new cutoff with time to AD-type dementia progression, including age and sex as covariates. For these analyses, nondemented patients were included when they had at least 6 months of follow-up available. We compared both models with chi-square tests of the log-likelihood ratio. A difference with a *p* value less than 0.05 was considered significant. Statistical analyses for multivariate Cox regression were performed using R version 3.2.3 software.

Finally, we studied the minimum number of subjects per clinical population necessary to reliably estimate the cutoff in a data-driven way. To this end, we simulated CSF Aβ_42_ values from a bimodal distribution with mean and SD values as estimated from our dataset. We recalculated the cutoffs and 95% CIs for sample sizes with varying numbers starting from *n* = 300 to 3000 with steps of 100. The minimum sample size required to obtain a reliable cutoff was determined as the sample size for which 95% CI lines were larger than the mean cutoff ±10%, which is currently used as a rule-of-thumb indication of acceptable variability in CSF Aβ_42_ levels.

## Results

### Baseline characteristics

Table 1 shows the baseline characteristics according to diagnostic group. Briefly, patients with AD and patients with MCI were older and included a higher percentage of APOE ε4 allele carriers than the other groups. CSF Aβ_42_ levels were highest for SCD, followed by non-AD-type dementia, MCI, and AD-type dementia. Of the nondemented subjects, over an average follow-up period of 3.2 (SD 2.04) years, 21 (9%) of the subjects with SCD progressed to MCI and 13 (5%) to AD-type dementia, and 146 (39%) of the subjects with MCI progressed to AD-type dementia.

**Table 1:** Subject characteristics

	a. All (*n* = 2462)	b. SCD (*n* = 448)	c. MCI (*n* = 490)	d. AD-type dementia (*n* = 1031)	e. Non-AD-type dementia (*n* = 493)
Age, years	66.8 (7.0)	64.4 (6.2)^b,c,d^	68.2 (6.9)^a,c,d^	67.3 (7.2)^a,b^	66.9 (6.8)^a,b^
Female sex, *n* (%)	1049 (43)	170 (38)^c^	181 (37)^c^	528 (51)^a,b,d^	170 (34)^c^
Years of education	11.1 (3.0)	11.9 (3.1)^b,c,d^	11.4 (3.2)^a,c,d^	10.8 (2.8)^a,b^	10.5 (2.9)^a,b^
MMSE score	23.6 (5.2)	28.3 (1.7)^b,c,d^	26.5 (2.5)^a,c,d^	20.4 (5.0)^a,b,d^	23.0 (5.2)^a,b,c,^
APOE ε4 allele carriers, *n* (%)	1186 (54)	158 (35)^b,c^	242 (49)^a,c,d^	615 (60)^a,b,d^	171 (35)^b,c^
CSF Aβ_1–42_, pg/ml	667 (289)	906 (277)^b,c,d^	676 (295)^a,c,d^	504 (174)^a,b,d^	781 (278)^a,b,c^
CSF tau, pg/ml	527 (401)	317 (205)^b,c,d^	486 (313)^a,c,d^	705 (406)^a,b,d^	394 (443)^a,b,c^
CSF p-tau, pg/ml	70 (37)	52 (25)^b,c^	70 (35)^a,c,d^	88 (39)^a,b,d^	50 (25)^b,c^
Outcome at follow-up AD-type/no AD-type dementia (% AD-type dementia)	–	13/235 (5)	146/224 (61)	–	–
Average follow-up duration, years	–	2.93 (1.99)	2.41 (1.46)	–	–

Data are mean (SD), unless otherwise specified. Superscript letters indicate that this group shows a statistically significant difference (*p* < .05) with other groups as labelled with a,b,c,d or e in the column headers

*Abbreviations*: *SCD* Subjective cognitive decline, *MCI* Mild cognitive impairment, *AD* Alzheimer’s Disease, *APOE* Apolipoprotein E, *CSF* Cerebrospinal fluid, *Aβ*_*1–42*_ β-Amyloid 1–42, - Not applicable

### CSF Aβ_1–42_ cutoff based on mixture modeling

**Fig. 1:**
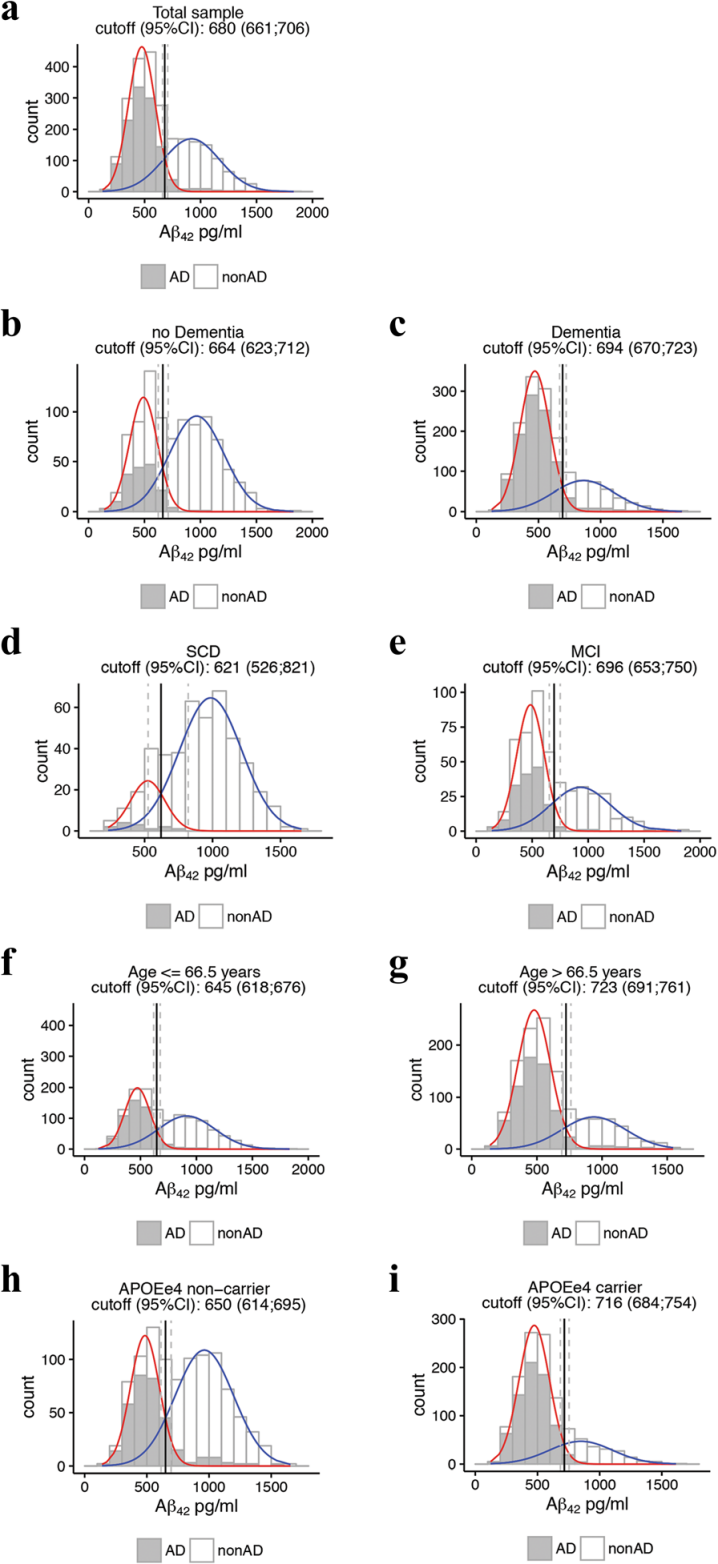
Cerebrospinal fluid β-amyloid 1–42 (Aβ_42_) cutoff values based on mixture modeling. **a** Total sample. **b** Demented subjects. **c** Nondemented subjects. **d** Subjects with subjective cognitive decline (SCD). **e** Subjects with mild cognitive impairment (MCI). **f** Subjects ≤66.5 years old. **g** Subjects >66.5 years old. **h** Apolipoprotein E (APOE) ε4 allele noncarriers. **i** APOE ε4 allele carriers. Subjects with a clinical diagnosis of Alzheimer’s disease (AD)-type dementia at baseline or at follow-up (nondemented subjects) are shown in *gray*

In the total sample and all subgroups, a bimodal distribution best fitted the data (Table 2). In the total sample, this yielded a cutoff of 680 pg/ml (95% CI 660–705 pg/ml) (Fig. 1a). With this cutoff, 55% of our population fell into the abnormal amyloid distribution. Similar cutoffs were found when we repeated mixture modeling within the dementia group (694 pg/ml, 95% CI 670–721 pg/ml), the pooled sample of subjects with SCD and MCI (664 pg/ml [95% CI 621-712 pg/ml), subjects with SCD (621 pg/ml (95% CI 526-901 pg/ml) and subjects with MCI (696 pg/ml [95% CI 654-758 pg/ml) (Fig. 1b–e). A lower cutoff was found for subjects younger than 66.5 years (645 pg/ml [95% CI 617-678 pg/ml) than subjects older than 66.5 years (723 pg/ml [95% CI 691-762 pg/ml) (Fig. 1f and g). The cutoff for CSF Aβ_42_ was higher in APOE ε4 carriers than in noncarriers (resp. 716 pg/ml [95% CI 684-756 pg/ml; 650 pg/ml [95% CI 611-689 pg/ml), but this did not reach statistical significance (Fig. 1h and i).

**Table 2:** Fit statistics from bootstrap to test the null hypothesis of a *K*-component fit versus (*K* + 1)-component fit for total sample

	Log-likelihood 1 versus 2 components			Log-likelihood 2 versus 3 components		
	Observed	Bootstrap (95% CI)	*p* Value	Observed	Bootstrap (95% CI)	*p* Value
All subjects	653.98	6.51 (1.65–15.69)	<0.001	2.16	16.67 (0.78–11.15)	0.77
Subjective cognitive decline	15.64	6.12 (1.72–13.25)	0.03	2.45	5.8 (0.94–17)	0.74
Mild cognitive impairment	124.50	5.79 (1.91–12.63)	<0.001	9.95	5.55 (0.54–10.82)	0.08
Nondemented	132.93	6.44 (1.5–13.1)	<0.001	10.41	4.34 (0.73–11.3)	0.07
Dementia	471.81	6.01 (1.49–13.21)	<0.001	3.61	4.34 (0.53–9.56)	0.55
Younger than 66.5 years old	261.16	5.86 (1.4–12.82)	<0.001	2.02	4.64 (0.65–9.83)	0.79
Older than 66.5 years old	397.49	6.42 (1.82–14.46)	<0.001	9.39	4.82 (0.68–11.64)	0.11
APOE ε4 noncarrier	138.75	6 (1.77–13.01)	<0.001	4.19	6.91 (0.79–11.26)	0.41
APOE ε4 carrier	367.29	5.83 (1.89–11.50)	<0.001	10.49	7.25 (0.6–11.79)	0.07

*APOE* Apolipoprotein E

### Comparison with previously defined cutoff

**Fig. 2:**
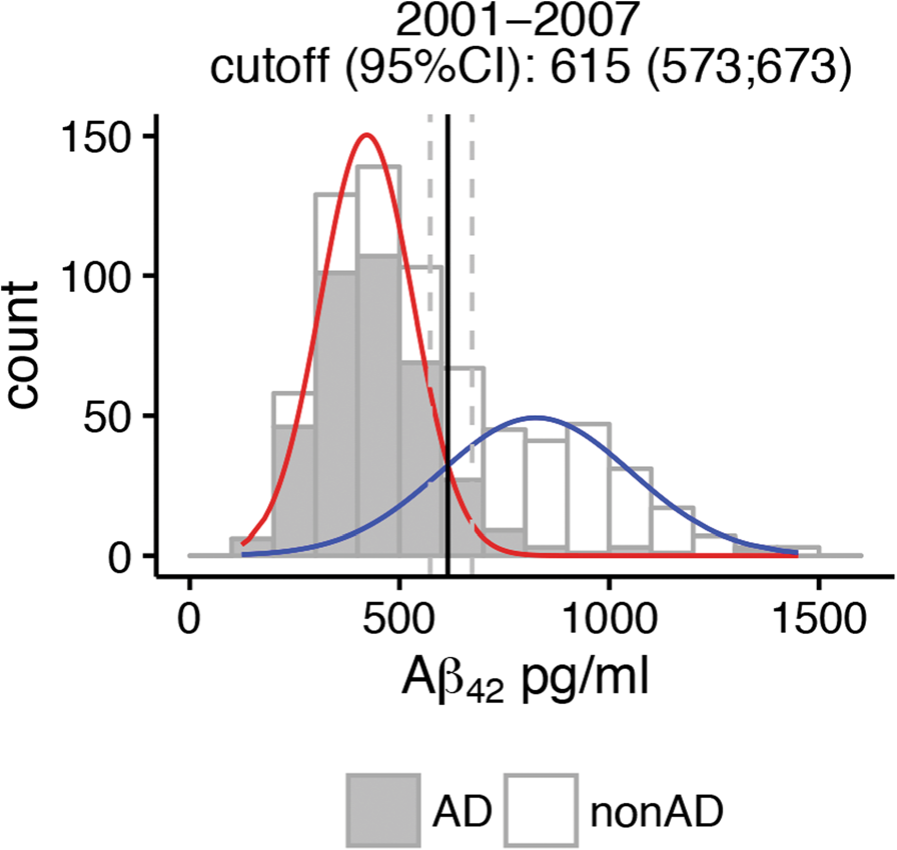
Cerebrospinal fluid β-amyloid 1–42 (Aβ_42_) cutoff values based on mixture modeling in subjects seen between 2001 and 2007. Subjects with a clinical diagnosis of Alzheimer’s disease (AD)-type dementia at baseline or at follow-up are shown in *gray*

The cutoff of 680 pg/ml based on mixture modeling was substantially higher than our previously clinically defined cutoff of 550 pg/ml (95% CI 531–570 pg/ml) [11]. Repeated mixture modeling in the subset of subjects with CSF analysis in the 2001–2007 period (in which subjects were selected for the clinically based cutoff calculation) also resulted in a higher cutoff (615 pg/ml, 95% CI 573–673 pg/ml) (Fig. 2) than the clinically defined cutoff.

In this subset, using a cutoff value of 615 pg/ml, 439 subjects were classified as having abnormal amyloid, which was a 13% increase compared with the 390 subjects classified by the clinically defined cutoff. The sensitivity of the cutoff of 615 pg/ml for AD-type dementia was 0.89 with a specificity of 0.62. The clinically defined cutoff of 550 pg/ml resulted in a sensitivity of 0.86 with a specificity of 0.65. Of the nondemented subjects who later progressed to AD-type dementia, 87% had CSF Aβ_42_ levels less than 615 pg/ml, compared with 76% with CSF Aβ_42_ levels less than 550 pg/ml. For nondemented subjects who did not progress to AD-type dementia, these proportions were 30% versus 21%, respectively (Table 3). Survival analyses showed that both cutoffs were predictive of the time to development of AD-type dementia (550 pg/ml cutoff HR = 5.14, 95% CI 2.96–8.93; pg/ml; versus 615 pg/ml cutoff HR 7.44, 95% CI 3.74–14.79) (Table 4). The HR for the development of AD-type dementia was significantly greater for the new cutoff of 615 pg/ml than for the cutoff of 550 pg/ml (*p* < 0.001).

**Table 3:** Number of subjects of subsample between 2001 and 2007 according to diagnosis at baseline, outcome at follow-up, and cerebrospinal fluid β-amyloid 1–42 cutoff score

	Total group (*n* = 688)	CSF Aβ_42_ cutoff <550 pg/ml	CSF Aβ _42_ cutoff <615 pg/ml
AD-type dementia, *n* (%)	288 (42)	236 (82)	255 (89)
Non-AD-type dementia, *n* (%)	143 (21)	49 (34)	55 (38)
SCD with follow-up available			
Converted to AD-type dementia, *n* (%)	7 (7)	5 (71)	6 (86)
Not converted to AD-type dementia, *n* (%)	91 (93)	16 (18)	20 (22)
MCI with follow-up available			
Converted to AD-type dementia, *n* (%)	69 (53)	53 (77)	60 (87)
Not converted to AD-type dementia, *n* (%)	60 (47)	16 (27)	26 (43)

Data are from the subsample of patients seen between 2001 and 2007. For this time period, the cutoff for CSF Aβ _42_ determined with Gaussian mixture modeling was 615 pg/ml

*Abbreviations*: *CSF* Cerebrospinal fluid, *Aβ*_*42*_ Amyloid-β 1–42, *SCD* Subjective cognitive decline, *MCI* Mild cognitive impairment, *AD* Alzheimer’s disease

**Table 4:** Cox proportional HRs (95% CIs) for clinical progression to Alzheimer’s disease-type dementia in nondemented subjects

CSF Aβ_42_ cutoff	HR (95% CI)	Log-likelihood ratio	χ^2^
<550 pg/ml	5.14 (2.96–8.93)^a^	−327.85	n.a.
<615 pg/ml	7.44 (3.74–14.79)^a^	−324.66	6.38^b^

Analyses were adjusted for age and sex. Data are from the subsample of nondemented sample with at least 6 months follow-up available seen between 2001 and 2007. The event rate was 33%, and about 13% of subjects were lost to follow up per year

*Abbreviations*: *CSF* Cerebrospinal fluid, *Aβ*_*42*_ β-Amyloid 1–42, *n.a.* Not applicable

^a^ *p* = 0.0001

^b^Decrease in χ^2^ *p* < 0.01

We further explored why the data-driven cutpoint was somewhat lower in the subset of subjects with CSF analysis in the 2001–2007 period than in the total sample. Additional file 1: Figure S1 shows that the peaks of CSF Aβ_42_ level distributions seem to shift over subsequent years. This could not be explained by a difference in patient population, because the distribution of diagnoses remained comparable over time (χ^2^ (45) = 61.32, *p* > 0.05) (Additional file 1: Table S1). We further explored whether this shift was due to an assay drift, and we repeated all subgroup analyses stratified for the time period when the lumbar puncture was obtained (2001–2007 versus 2008–2015) (see Additional file 1: Table S2 for baseline characteristics). Briefly, the cutpoint in the total group was higher in the 2008–2015 subsample (697 pg/ml [675–723 pg/ml]) than in the 2001–2007 subsample (615 pg/ml [573–673 pg/ml]) (Additional file 1: Table S3 and Figure S2 and S3). Subgroup analyses showed that the cutpoint for the dementia group and for APOE ε4 allele carriers was also higher in the 2008–2015 group than in the 2001–2007 group. Cutpoints for other subgroups did not differ between time periods.

### Minimum sample size required

Additional file 1: Figure S4 shows the mean and SD values for different sample sizes, varying from 300 to 3000 subjects with steps of 100. The average CSF Aβ_42_ cutoff was 679 pg/ml and remained similar with increasing sample size, whereas the 95% CI became narrower. Accepting a maximum deviation from the cutoff of ±10%, a minimum sample size of 800 is required for an acceptable 95% CI of 637–744 pg/ml. Our determined cutoff of 680 pg/ml and 95% CI fell within this range.

## Discussion

Using a data-driven Gaussian mixture modeling approach, we determined a cutoff of 680 pg/ml for abnormal CSF Aβ_42_ levels. This cutoff was independent of the cognitive stage and APOE genotype. The cutoff was higher in older than in younger subjects. With this new cutoff, a good classification of subjects with underlying AD pathology was achieved because 88% of nondemented subjects who later developed AD-type dementia had CSF Aβ_42_ levels below our new cutoff. Our results suggest that mixture modeling is a robust method to determine cutoff values for CSF Aβ_42_.

In the total sample, we found that subjects with AD-related characteristics (dementia, MCI, older age, and APOE ε4 carriers) fell mainly within the abnormal amyloid distribution and that subjects without AD-related characteristics (SCD, younger age, and APOE ε4 noncarriers) fell mainly within the normal amyloid distribution. This supports the idea that a bimodal distribution of amyloid levels represents normal and abnormal distributions of amyloid. When comparing cutoffs based on age and APOE ε4 groups, the data-driven cutoff was higher in older than in younger subjects and tended to be higher in APOE ε4 allele carriers than in noncarriers. One explanation for the higher cutoff in these groups is that it reflects a difference in amyloid processing. However, this is unlikely, because a previous study showed that CSF Aβ_42_ levels were not dependent on APOE ε4 genotype after correction for Aβ deposition as measured by amyloid positron emission tomography (PET) [22]. Furthermore, researchers in another study found a similar cutoff for CSF Aβ_42_ across age groups [23]. A more likely explanation for the higher cutoff is that, within the old and APOE ε4 groups, relatively few subjects had normal CSF Aβ_42_ levels. As a consequence, the distribution of values reflecting normal Aβ_42_ levels was relatively wide, which resulted in a shift to a higher cutoff that separated the abnormal and normal CSF Aβ_42_ distribution. Thus, the variability in cutoffs is more likely to result from different sampling frequencies of normal and abnormal Aβ_42_ populations rather than from differences in amyloid processing. This suggests that, in order to determine a cutoff with this data-driven approach, the data need to contain a sufficient sample from both the normal and abnormal populations. For example, for the SCD group, which had few subjects who had abnormal CSF Aβ_42_, the 95% CI was wide, so the resulting cutpoint should be considered with caution.

The cutoff of 680 pg/ml defined by Gaussian mixture modeling is higher than our previous clinically defined cutoff of 550 pg/ml. This indicates that clinically based cutoffs may underestimate the presence of abnormal CSF Aβ_42_. Still, the difference in cutoffs may also have resulted from drift in CSF Aβ_42_ levels over time, owing to variability in batches used for the biomarker analysis [6]. To test this possibility, we repeated our analyses based on data obtained in a time period similar to the previously clinically defined cutoff (2001–2007). Indeed, the cutpoint in the 2001–2007 subsample (615 pg/ml) was lower than that in the 2007–2015 subsample (697 pg/ml). Nevertheless, the data-driven cutoff derived for the 2007–2015 subsample was still higher than the clinically defined cutoff of 550 pg/ml, suggesting that our higher cutoff value was not due simply to a change in assay performance over time.

Our data-driven cutoff is within the same range as the CSF Aβ_42_ cutoff that shows the best concordance with amyloid PET (640 pg/ml) in our cohort [24] and in other cohorts (616–647 pg/ml) in which CSF Aβ_42_ was assessed with the Innotest assay [25–27]. This similarity between the amyloid PET derived cutoff for CSF Aβ_42_ and our new cutoff suggests that Gaussian mixture modeling is better able than a cutoff based on clinical diagnosis to capture and differentiate subjects from a memory clinic sample in terms of amyloid pathology.

The higher cutoff led to an increased sensitivity to detect subjects with AD-type dementia at baseline and an increased sensitivity to predict future AD-type dementia in subjects with SCD or MCI. This increase in sensitivity could at least in part explain the results of a recent study that demonstrated an increased risk for cognitive decline in subjects with low normal CSF Aβ_42_ values, based on a classical clinically defined cutoff [28]. However, subjects with a non-AD type dementia and subjects with SCD and MCI who did not convert to AD-type dementia also more often had abnormal CSF Aβ_42_ with the new cutoff. Some of these subjects may have low normal scores, but it could also mean that amyloid positivity is typically underestimated in these populations. For example, it is possible that some of the subjects with SCD and MCI might have developed AD-type dementia after the period in which they were followed.

A strength of our study is the availability of a large, clinically well-characterized cohort with longitudinal data. This made it possible to analyze cutoffs for CSF Aβ_42_ for different subgroups of patients and to assess the ability of the new cutoff to detect future AD-type dementia before cognitive impairment becomes evident. A limitation of the Cox regression analysis was that the time to dementia was an approximation because the event occurred at an unknown time between two visits.

The best gold standard currently available to measure amyloid pathology in vivo is amyloid PET because this correlates strongly with amyloid status determined postmortem [29]. The similarity in cutoffs between our approach and amyloid PET indicates that centers that do not have PET techniques available can improve the accuracy of detecting abnormal amyloid with mixture modeling. A possible limitation of the method, however, is that the sample used for the cutoff definition should contain a sufficient number of subjects with normal and abnormal amyloid. We found that at least 800 subjects are needed to obtain a sufficiently reliable cutoff for CSF Aβ_42_. This number was calculated under the assumption that, in a memory clinic (as in ours), 55% of patients have abnormal CSF Aβ_42_ levels and 45% have normal levels. However, these distribution parameters might not apply to all memory clinics, because they are dependent on the type of patients seen and their age. For example, if 60% of patients in a population had abnormal CSF Aβ_42_ levels and 40% had normal CSF Aβ_42_ levels, a sample size of at least 450 subjects would be needed to determine a reliable cutoff (data not shown).

## Conclusions

Using a data-driven mixture method, we found a new cutoff for abnormal CSF Aβ_42_ levels that was higher and was better able to predict future AD-type dementia than our clinically determined cutoff. The increase in cutoff for CSF Aβ_42_ has implications for clinical practice because more patients will be labeled as having AD pathology than were so labeled using the old cutoff. This is likely to impact communication with and management of patients [30]. Still, regardless of the cutoff used at this time, the decision whether to communicate abnormal Aβ_42_ values with patients will be challenging because no therapy is yet available. Disclosure of pathological diagnosis will probably depend on the AD stage, the wish of patients to know, and the view of the clinician regarding this topic [31].

## Additional file

Additional file 1: Comparison with previously defined cutoff. (PDF 2355 kb)

## Abbreviations

AD: Alzheimer’s disease
ADC: Amsterdam Dementia Cohort
APOE: Apolipoprotein E
Aβ: β-Amyloid
CSF: Cerebrospinal fluid
MCI: Mild cognitive impairment
MMSE: Mini Mental State Examination
PET: Positron emission tomography
p-tau: Phosphorylated tau
SCD: Subjective cognitive decline
