# Supplementary material for: Unbiased estimates of cerebrospinal fluid β-amyloid 1–42 cutoffs in a large memory clinic population
Source: Alzheimers Res Ther. 2017 Feb 14;9:8. doi: 10.1186/s13195-016-0233-7 (PMC5307885; doi:10.1186/s13195-016-0233-7)
Supplement: Additional file 1: — Comparison with previously defined cutoff. (PDF 2355 kb) [file 13195_2016_233_MOESM1_ESM.pdf]

Unbiased estimates of CSF beta amyloid 1-42 cut-off in a large memory clinic population

Daniela Bertens, MD; Betty M. Tijms, PhD; Philip Scheltens MD, PhD; Charlotte E. Teunissen PhD; Pieter Jelle Visser MD, PhD.

Supplementary tables and figures

Supplementary Table 1. Distribution of diagnosis per year of CSF collection

Year	Subjective complaints	MCI	Probable AD	Dementia other
2001	4 (11.76%)	12 (35.29%)	11 (32.35%)	7 (20.59%)
2002	8 (13.33%)	17 (28.33%)	26 (43.33%)	9 (15%)
2003	11 (13.10%)	24 (28.57%)	37 (44.05%)	12 (14.29%)
2004	17 (20%)	18 (21.18%)	30 (35.29%)	20 (23.53%)
2005	10 (10.42%)	20 (20.83%)	39 (40.63%)	27 (28.13%)
2006	20 (13.70%)	32 (21.92%)	62 (42.47%)	32 (21.92%)
2007	38 (20.77%)	26 (14.21%)	83 (45.36%)	36 (19.67%)
2008	37 (17.79%)	40 (19.23%)	95 (45.67%)	36 (17.31%)
2009	50 (19.46%)	51 (19.84%)	100 (38.91%)	56 (21.79%)
2010	38 (20%)	35 (18.42%)	79 (41.58%)	38 (20%)
2011	68 (25.56%)	58 (21.80%)	98 (36.84%)	42 (15.79%)
2012	37 (14.98%)	51 (20.65%)	101 (40.89%)	58 (23.48%)
2013	50 (19.08%)	54 (20.61%)	112 (42.75%)	46 (17.56%)
2014	40 (19.23%)	31 (14.90%)	88 (42.31%)	49 (23.56%)
2015	19 (14.84%)	21 (16.41%)	66 (51.56%)	22 (17.19%)

Supplementary Table 2. Baseline characteristics according to time of CSF collection

2001-2007	All (n=654)	SCD (n=104)	MCI (n=137)	AD-type dementia (n=277)	Non-AD type dementia (n=136)
Age (years)	68.3 (8.0)	64.8 (7.5)	70.1 (7.4)	68.9 (8.1)	68 (7.8)
Females (%)	360 (55%)	64 (62%)	82 (60%)	133 (48%)	81 (60%)
Years of education	4.8 (1.4)	5.3 (1.4)	4.8 (1.4)	4.7 (1.4)	4.6 (1.5)
MMSE score	23.7 (5.2)	28.4 (1.8)	26.5 (2.7)	21.1 (4.9)	22.8 (5.8)
APOE-ε4 allele carriers, n (%)	308 (47%)	36 (35%)	59 (43%)	174 (63%)	39 (29%)
CSF aß 1-42 (pg/ml)	592 (257)	796 (237)	581 (251)	463 (169)	711 (274)
CSF tau (pg/ml)	563(397)	327 (236)	546 (398)	728 (418)	429 (295)
CSF ptau (pg/ml)	74 (39)	52 (23)	75 (40)	92 (39)	55.15 (27)

2008-2015	All (n=1766)	SCD (n=339)	MCI (n=341)	AD-type dementia (n=739)	Non-AD type dementia (n=347)
Age (years)	66.3 (6.5)	64.2 (5.8)	67.4 (6.5)	66.8 (6.6)	66.5 (6.3)
Females (%)	1029 (58%)	211 (62%)	222 (65%)	362 (49%)	234 (67%)
Years of education	5.0 (1.3)	5.4 (1.2)	5.2(1.4)	4.9 (1.3)	4.7 (1.4)
MMSE score	23.5 (5.3)	28.2 (1.7)	26.6 (2.4)	20.2 (5.0)	23.1 (5.0)
APOE-ε4 allele carriers, n (%)	853 (48%)	120 (35%)	175 (51%)	430 (58%)	128 (37%)
CSF aß 1-42 (pg/ml)	700 (294)	942 (280)	722 (301)	525 (172)	815 (273)
CSF tau (pg/ml)	513 (401)	313 (194)	456 (265)	698 (400)	380 (493)
CSF ptau (pg/ml)	69 (37)	52 (25)	67 (33)	87 (39)	49 (24)

Abbrevations: SCD, subjective cognitive decline; MCI, Mild Cognitive Impairment; AD, Alzheimer's Disease; APOE, Apolipoprotein E; CSF, cerebrospinal fluid; aß 1-42, amyloid beta 1-42, n.a. not applicable. Data are mean (SD), unless otherwise specified.

Supplementary Table 3. Cut-point according to period of CSF collection

Year	All (n=2462)	2001-2007 (n=654)	2008-2015 (n=1766)
a. All	680 (661-706)	615 (573-673)	697 (675-723)*
b. No dementia	664 (623-712)	696 (578-863)	672 (628-731)
c. Dementia	694 (670-723)	610 (567-657)[§]	715 (687-743)*
d. SCD	621 (526-821)	761 (549-1015)	638 (538-1136)
e. MCI	696 (653-754)	724 (593-834)	698 (653-773)
f. Age <= 66.5	645 (618-676)	644 (567-732)	666 (661-706)
g. Age >66.5	723 (691-761)[#]	651 (584-724)	737 (702-781)
h. APOE-e4 non-carrier	650 (614-695)	597 (529-697)	672 (633-712)
i. APOE-e4 carrier	716 (684-780)	586 (529-672)[§]	734 (696-780)*

* Statistically significantly different between 2001-2007 and 2008-2015 cut-off.
[§] Statistically significantly different between 2001-2007 and All cut-off.
[#] Statistically significantly different from Age <66.5 subgroup.

S-Figure 1: Distribution of CSF Aß42 values according to year of visit

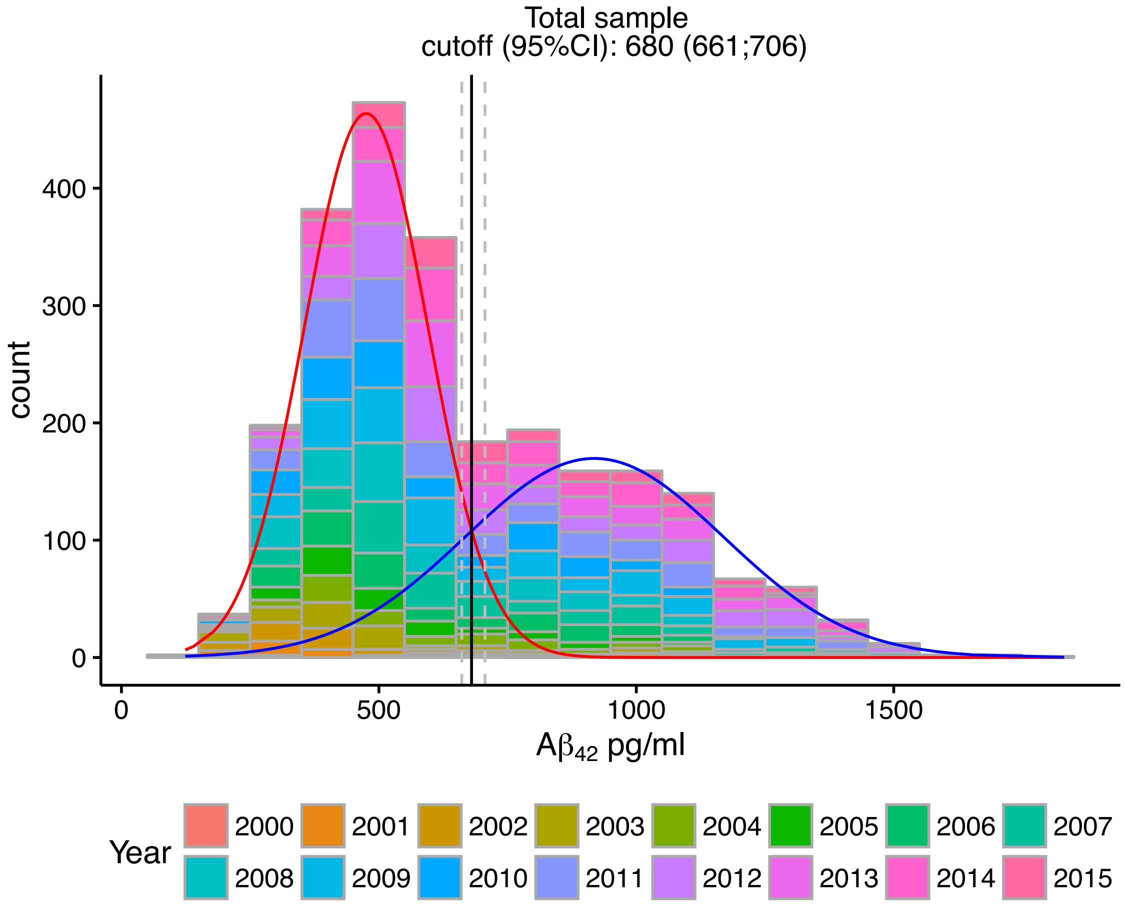

Colours represent the year that CSF samples were obtained.

S-Figure 2. CSF Aß42 cut-off values based on mixture modeling in 2001-2007 subsample

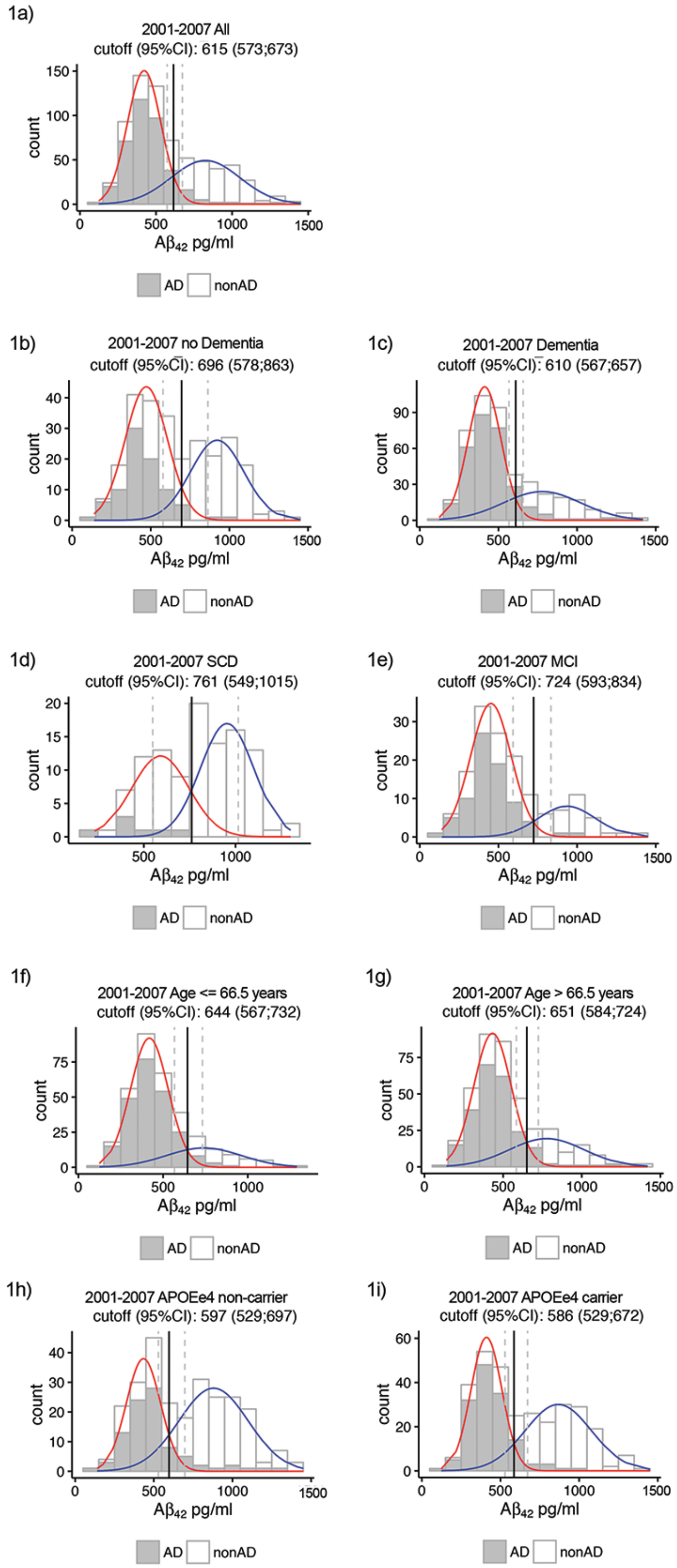

Legend S-Figure 2.

a) Total sample, b) Demented subjects, c) Non demented subjects, d) Subjects with SCD, e) Subjects with MCI, f) Subjects <= 66.5 years, g) Subjects > 66.5 years, h) APOE-ε4 allele non-carriers i) APOE-ε4 allele carriers. Subjects with a clinical diagnosis of AD-type dementia at baseline or at follow-up (non-demented subjects) are shown in grey.

Abbreviations: SCD, subjective cognitive decline; MCI, Mild Cognitive Impairment; APOE, Apolipoprotein E; AD, Alzheimer's disease.

S-Figure 3. CSF Aß42 cut-off values based on mixture modeling in 2008-2015 subsample

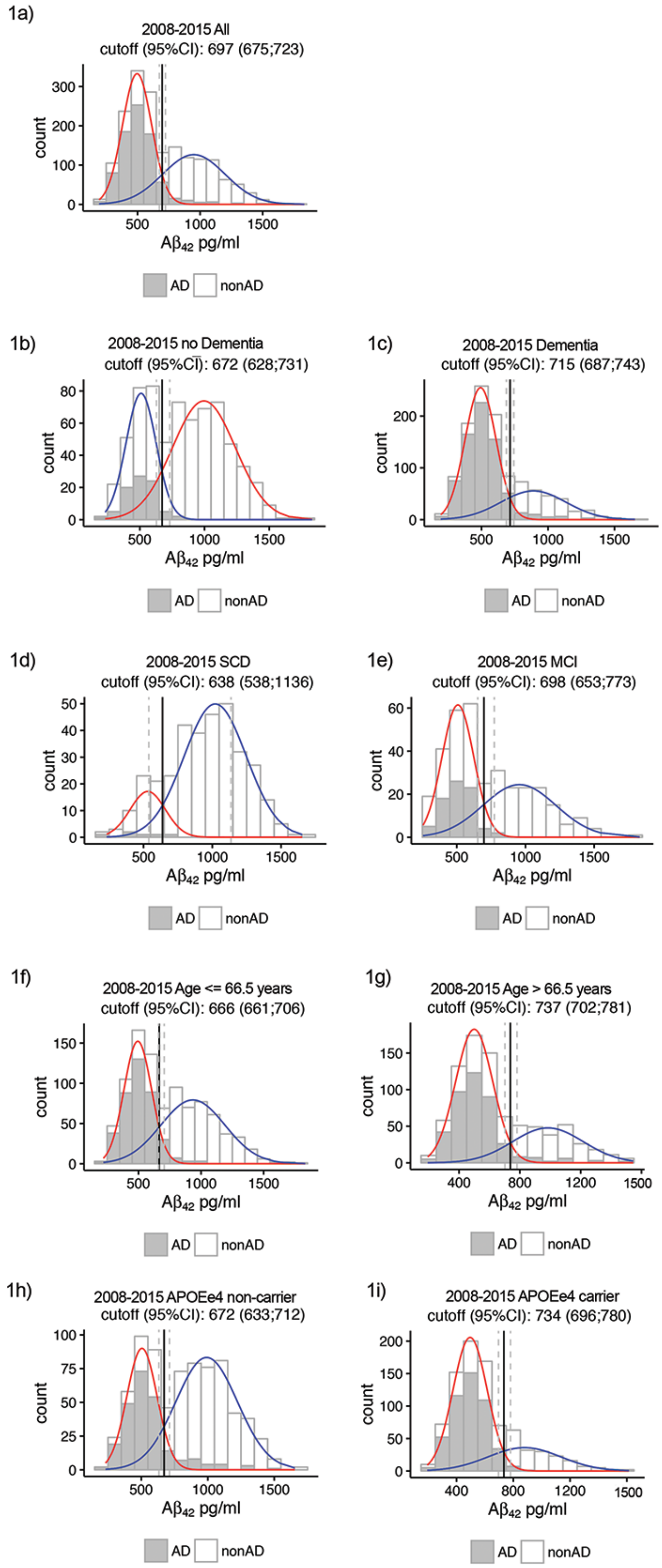

Legend S-Figure 3.

a) Total sample, b) Demented subjects, c) Non demented subjects, d) Subjects with SCD, e) Subjects with MCI, f) Subjects <= 66.5 years, g) Subjects > 66.5 years, h) APOE-ε4 allele non-carriers i) APOE-ε4 allele carriers. Subjects with a clinical diagnosis of AD-type dementia at baseline or at follow-up (non-demented subjects) are shown in grey.

Abbreviations: SCD, subjective cognitive decline; MCI, Mild Cognitive Impairment; APOE, Apolipoprotein E; AD, Alzheimer's disease.

S-Figure 4. Effect of sample size on CSF Aß 42 cut-off and 95% CI

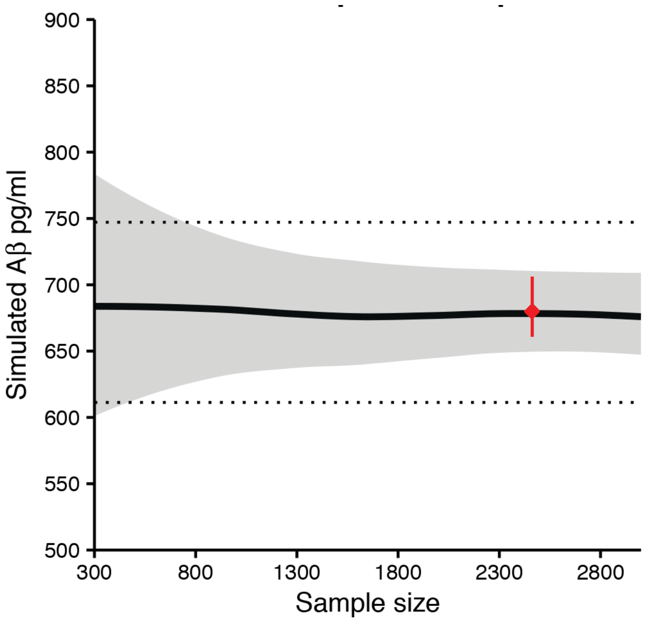

The solid black line represents the mean CSF Aß 1-42 cut-off per sample size and the grey area represents the 95% CI for that CSF aß 1-42 cut-off. The dotted lines represent the mean cut-off + or - 10%. The place were the 95% CI crosses the dotted line represents the minimum sample size required (here n=800) to obtain a cut-off of which the 95% CI is within 10% of the mean cut-off. Our data-driven cut-off with 95% CI is represented in red. The modeling was performed in population of which 55% had abnormal CSF aß 1-42 and 45% normal levels. Abbreviations: aß 1-42, amyloid beta 1-42.
